# Relativity Theory and Time Perception: Single or Multiple Clocks?

**DOI:** 10.1371/journal.pone.0006268

**Published:** 2009-07-22

**Authors:** Catalin V. Buhusi, Warren H. Meck

**Affiliations:** 1 Department of Neurosciences, Medical University of South Carolina, Charleston, South Carolina, United States of America; 2 Department of Psychology and Neuroscience, Duke University, Durham, North Carolina, United States of America; Istituto di Neurofisiologia, Italy

## Abstract

**Background:**

Current theories of interval timing assume that humans and other animals time as if using a single, absolute stopwatch that can be stopped or reset on command. Here we evaluate the alternative view that psychological time is represented by multiple clocks, and that these clocks create separate temporal contexts by which duration is judged in a relative manner. Two predictions of the multiple-clock hypothesis were tested. First, that the multiple clocks can be manipulated (*stopped* and/or *reset*) independently. Second, that an event of a given physical duration would be perceived as having different durations in different temporal contexts, i.e., would be judged differently by each clock.

**Methodology/Principal Findings:**

Rats were trained to time three durations (e.g., 10, 30, and 90 s). When timing was interrupted by an unexpected gap in the signal, rats *reset* the clock used to time the “short” duration, *stopped* the “medium” duration clock, and continued to *run* the “long” duration clock. When the duration of the gap was manipulated, the rats reset these clocks in a hierarchical order, first the “short”, then the “medium”, and finally the “long” clock. Quantitative modeling assuming re-allocation of cognitive resources in proportion to the relative duration of the gap to the multiple, simultaneously timed event durations was used to account for the results.

**Conclusions/Significance:**

These results indicate that the three event durations were effectively timed by separate clocks operated independently, and that the same gap duration was judged relative to these three temporal contexts. Results suggest that the brain processes the duration of an event in a manner similar to Einstein's special relativity theory: A given time interval is registered differently by independent clocks dependent upon the context.

## Introduction

Over a century ago, Albert Einstein postulated that a given time interval is registered differently by independent (moving) clocks [1]. Interestingly, Einstein himself recognized the similarity between the relativity of physical and psychological time: “When a man sits with a pretty girl for an hour, it seems like a minute. But let him sit on a hot stove for a minute - and it's longer than any hour. That's relativity.” [2] Einstein was literally talking about different temporal contexts providing different read-outs for the same physical interval. While relative time became the de-facto view in physics, the relativity of psychological time is still a matter for debate. For example, neurobiological evidence suggests indeed that major time scales (millisecond, second-to-minutes, and circadian) are processes by different regions of the brain (e.g., cortex, cerebellum, striatum, and suprachiasmatic nucleus) [3], it is still unclear whether everyday timing in the seconds-to-minutes range is performed by a single or rather multiple parallel mechanisms in the brain [4].

The prevalent view in cognitive theories of psychological time is that humans and other animals time multiple durations by using a single stopwatch with a clock stage composed of a pacemaker whose pulses are passed through a switch into an accumulator, the output of which is manipulated by simple arithmetic [5]. Although some procedures are amenable to timing with a single stopwatch that is used in a sequential manner, evidence for simultaneous temporal processing can be found even in relatively simple timing tasks [6]–[10]. For example, when rats are trained on a tri-peak procedure in which three levers are individually associated with different durations initiated by the onset of a single signal, the responses on the three levers demonstrate a high degree of independence, suggesting the use of independent clocks rather than a single stopwatch [11], [12]. This raises the question of whether subjects use a single stopwatch whose output is manipulated using simple arithmetic or multiple independent clocks in order to time multiple durations concurrently. Assuming for the moment a “pacemaker-switch-accumulator” model, it is an open question as to whether the same pacemaker could be used to generate pulses that are then switched into multiple accumulators or whether the observed independence would also require the equivalent of multiple pacemakers in addition to the multiple “switch-accumulator” components [13].

Here we investigate two direct predictions of the view that time is represented *relative to multiple independent clocks*: (a) that these independent clocks can be operated (e.g., stopped or reset) independently, and (b) that these clocks create separate temporal contexts by which any given duration is judged, such that the same duration is judged differently by each of these clocks, in line with Einstein's insight. We addressed these questions in a tri-peak procedure by challenging rats with unexpected retention-intervals (gaps).

When unpredictable, infrequent gaps interrupt a timed signal, subjects behave as if they *run*, *stop*, or *reset* their clocks [14]–[17]. In the run mode, subjects ignore the gap and continue timing, in the stop mode they retain the time prior to the gap and resume timing after the gap, and in the reset mode the time prior to the gap is lost, and subjects re-start timing after the gap anew. In the tri-peak procedure, we expected gaps to reveal the use of a single master clock or of multiple independent clocks. Should subjects use the same rule on all three durations it would implicate a single stopwatch; should they use different rules on the three durations—say run for one duration, stop for the second, and reset for the third—it would implicate separate clocks that can be manipulated independently, because such an output can not be obtained by manipulating the output of a single clock whose sequential operation would lack independence. We sought separate measures of independence of these putative clocks by analyzing the average response rates over several trials, as well as the pattern of responding during individual trials. During individual trials, the times when subjects switch from a low-state to a high-state of responding, *s_1_*, and from a high-state to a low-state of responding, *s_2_*, the difference in these times, *d* = *s_2_*−*s_1_*, and the midpoint of these times, *m* = (*s_2_*+*s_1_*)/2, have all been shown to be valuable in elucidating properties of the interval-timing system [18], [19]. Despite extensive investigation of the relationship between these statistics for a single target duration [19]–[21], there has not yet been an evaluation of the relationship of these variables across multiple durations. Of interest for our study were the interactions between these statistics across durations, and whether they are consistent with a single clock, or with multiple independent clocks.

A second major goal of this study was to evaluate whether the effect of a gap is dependent on its *absolute* duration – as predicted by the use of a single stopwatch – or on its *relative duration to the temporal context* in which the gap is presented [22]. For example, in a delay-matching-to-sample-duration (DMTS-D) procedure, the response chosen by pigeons when presented with a test interval depends on the relative duration of the test interval to the temporal context in which its is embedded [22], which includes (in the DMTS-D procedure) the delay after the test interval [23] as well as the inter-trial interval at the time of the test [24]. Therefore, by having rats time in parallel three durations each of which acting as a separate temporal context, we expected the effect of the gap to depend not on its absolute duration, but on its duration relative to the temporal context in which it is presented, i.e., relative to each temporal criterion.

To account for the effect of the gap in the tri-peak procedure, we extended the *qualitative description* of the relative-duration hypothesis [22] to a *quantitative model* of the effect of retention-intervals on timing [25]–[27]. Current data provide strong support for a *resource allocation* model [28] suggesting that the presentation a gap determines resources to be taken away from the timing system, such that, left with fewer resources, the timing system fails to maintain the current subjective time in working memory (WM), resulting in a delay in timing [25]–[29]. The model addresses manipulations of gap position, duration, and salience [8], [14]–[17], [25]–[27], [30]–[35] by assuming that during the gap resources decay (are re-allocated) in proportion to the *relative saliency* of the gap to the timed signal [25]–[27]. Here we extended this model to include the *relative duration* of the gap to the timed signal. To further examine the latter possibility, we conducted quantitative modeling assuming three independent clocks whose resources are independently re-allocated in proportion to the *relative duration* of the gap to the timed signals.

## Results

### Response Rate Analysis

**Figure 1:**
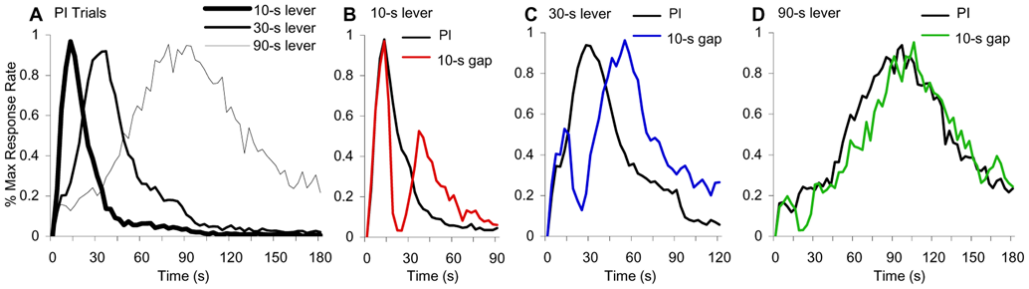
Response functions during probe trials of a tri-peak procedure (A) and during a 10-s gap trial: (B) 10-s lever, (C) 30-s lever, (D) 90-s lever.

Baseline tri-peak response functions peaked around the to-be-timed 10, 30, and 90-s criteria showing that rats acquired the tri-peak procedure, F(2,18) = 660.36, p<0.001 (Fig. 1A). Presentation of gaps inserted into the timed signal at a constant location, 15s from trial onset, differentially affected the response functions for the 3 durations: a 10-s gap delayed by approximately 30s the response function on the 10-s lever (reset rule, Fig. 1B), by approximately 20s the response on the 30-s lever (intermediate between stop and reset, Fig. 1C), and by approximately 10s the response on the 90-s lever (stop rule, Fig. 1D), F(4,36) = 122.84, p<0.001. The pattern of differential effects of the same gap on the 3 criterion durations suggests that the clocks associated with these durations can be manipulated independently. The effect of a gap seems to increase with its relative duration to each criterion: the effect was larger for the shorter criterion (reset, Fig. 1B) than for the longer criterion (stop, Fig. 1D).

**Figure 2:**
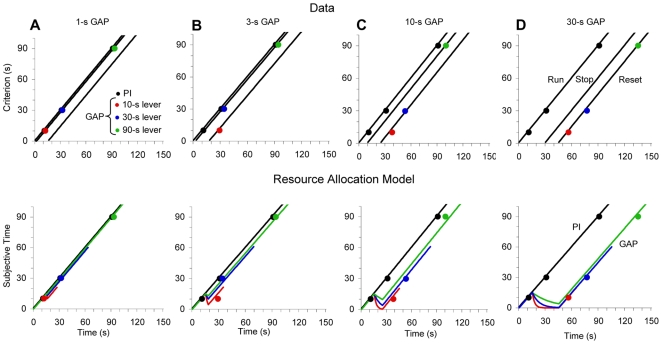
Data: Observed peak times in probe trials (black dots) and gap trials (colored dots). *Model:* Quantitative modeling with the resource allocation model. A: 1-s gap, B: 3-s gap, C: 10-s gap, D: 30-s gap. Red: 10-s clock, blue: 30-s clock, green: 90-s clock.

To dissociate whether the effect of the gap is associated with *independent clocks* or rather with *independent responses*, we evaluated whether manipulating the length of the gap changes the response rules for each criterion/lever or whether response rules are fixed for each lever. Plotting the obtained peak times for each criterion as a function of the gap duration revealed a pattern of hierarchical interaction between gap duration and criterion duration, F(8,72) = 3.16, p<0.01 (Fig. 2A-Data). A gradual increase in gap duration resulted in a gradual reset at all temporal criteria, in an orderly, hierarchical fashion: A 1-s gap minimally interfered with timing (Fig. 2A-Data), a 3-s gap reset the 10-s clock, but not the 30-s and 90-s clocks (Fig. 2B-Data), a 10-s gap reset both the 10-s and 30-s clocks, but did not affect the 90-s clock (Fig. 2C-Data), while a 30-s gap reset all clocks (Fig. 2D-Data). This pattern or response in which subjects tend to reset after longer gaps was reported previously [5], [14]–[17], [30], [36] and suggests that the run/stop/reset rules are not associated with the responses (i.e., pressing on the 3 levers), but rather associated with specific clocks.

### Individual-Trials Analysis

**Figure 3:**
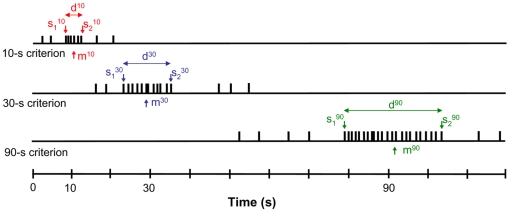
An example of the distribution of responses on the three levers (criteria) on an individual probe trial and associated statistics. Responses (vertical dashes) are shown for each criterion (10, 30, and 90 s) as a function of time. Color (red, blue, green) and superscripts indicate these measures for the 10, 30, and 90-s target durations, respectively.

An example of the distribution of responses on the three levers (criteria) on an individual probe trial is shown in Figure 3. Data from individual trials were analyzed to evaluate the following statistics: *s_1_*, defined as the time the high response state began for a particular target duration; *s_2_*, defined as the time that the high response state ended; the difference of these times, *d* = *s_2_*-*s_1_*, and the midpoint of these times, *m* = (*s_2_*+*s_1_*)/2. These statistics have all been shown to be valuable in elucidating properties of the internal clock [17], [18].

Strong positive correlations were found for the *s_1_*∶*s_2_* comparison within each criterion (0.56 to 0.69), as well as for the *s_1_*∶*m* and the *s_2_*∶*m* comparisons within each criterion duration (0.83 to 0.95). A strong negative correlation was also found for the *s_1_*∶*d* comparison within each criterion duration (−0.39 to −0.46). This pattern of correlations has been reported previously for different groups of rats trained on a peak-interval procedure [18]. In contrast, correlations of measures among criteria were considerably weaker. Weak positive correlations were found for the following comparisons: *s_1_^10^*∶*s_1_^30^* (0.15), *s_1_^10^*∶*s_1_^90^* (0.17), *s_2_^10^*∶*s_1_^30^* (0.12), *s_1_^10^*∶*m^30^* (0.10), *s_1_^10^*∶*m^90^* (0.11), and *s_2_^10^*∶*m^30^* (0.12). All correlations reported are significant at p<0.01; no other correlations were significant. The pattern of strong correlations for the parameters describing individual-trial behavior within each temporal criterion, and weak correlations among these parameters for the various pairs of temporal criteria, supports the proposal that independent clocks simultaneously timed the three durations.

### Quantitative Modeling

A resource allocation model of interval timing has been previously proposed, suggesting that gaps determine resources to be taken away from the timing system [25]–[27]. Previous implementations assumed that accumulated time decays exponentially during the gap at a rate proportional to the salience of the distracter relative to the timed signal. Here we extended this model by assuming that the decay rate is proportional to the relative duration of the gap to each criterion duration, -*salience* * *g*/*T_k_*. The relative salience of the gap is considered constant in the present simulations, but has been shown to be critical in addressing previous data sets [25], [26], [35]. If the criterion duration is short, the *g*/*T_k_* ratio is large, and working memory (WM) decays fast during the gap (reset rule); if the criterion duration is long, the *g*/*T_k_* ratio is small, and WM decays slowly (run or stop rule). Accordingly, simulations indicate that gaps determine differential effects on each clock, e.g., a 10-s gap resets the 10-s clock (Fig. 2C-Model red curve), delays the 30-s clock intermediate between stop and reset (Fig. 2C-Model blue curve), and stops the 90-s clock (Fig. 2C-Model green curve). Moreover, increasing the duration of the gap resets each clock in a hierarchical manner, first the 10-s clock (Fig. 2B-Model), then the 30-s clock (Fig. 2C-Model), and finally the 90-s clock (Fig. 2D-Model). These simulations quantitatively and qualitatively account for the current data and provide support for multiple independent clocks whose resources are dynamically re-allocated based on the relative duration of the gap to each temporal criterion.

## Discussion

We found that subjects simultaneously time different intervals using multiple independent clocks whose resources are independently re-allocated. When an infrequent, unpredictable gap interrupted the timed signals (Fig. 1*A*), rats reset the short clock, stopped the middle clock, and ran the long clock (Fig. 1B-D) suggesting that the three clocks were operated independently. Such temporal control of behavior can not be accounted for by a single clock whose output is manipulated by simple arithmetic. Moreover, when the duration of the gap was gradually increased, rats gradually reset their clocks at all three durations, in a hierarchical manner, first the short- (Fig. 2B-Model), then the middle- (Fig. 2C-Model), and lastly the long-criterion duration (Fig. 2D-Model), indicating clock-specific rather than response-specific operation. A resource allocation model accounted for the data under the assumption that the multiple clocks use resources which are independently re-allocated during the gap at rates proportional to the ratio *g*/*T_k_* between the duration of the gap, *g*, and the criterion of each clock, *T_k_* (Fig. 2A:D-Model).

### Implications for Psychological Models of Interval Timing

Several models have been previously applied to interpret data obtained in interval-timing experiments involving gaps (retention-intervals). A *switch* model, assuming that during the gap time fails to accumulate due to the opening of a stimulus-controlled switch, predicts that, irrespective of gap and criterion durations, subjects should use a stop rule [14], [15], [30], [31]. In contrast, the present data indicate that rats flexibly change their behavior with both gap and criterion duration. An *instructional-ambiguity* model proposing that subjects perceive the gap as an ambiguous, ITI-like event, predicts that manipulations that render the gap similar to the ITI should reset the clock, while manipulations that render the gap dissimilar from the ITI should stop the clock [37]. Because in our experiment the gap and ITI were identical (dark) this hypothesis predicts the use of the same rule (reset) at all gap and criteria durations, in contrast to the current data showing the use of different rules for different temporal criteria. To account for the flexible use of the run/stop/reset rules by rats and pigeons, Cabeza de Vaca et al. [38] proposed a *passive memory-decay* model which assumes that subjective time—stored in WM—decays passively during the gap. This model predicts that the effect of a gap should depend solely on absolute gap duration, but not on criterion duration, as found here (Fig. 1B-D). These models can not account for the differential effects of gaps on multiple intervals.

In contrast to these alternatives, the present *resource allocation* model assumes that during the gaps resources are re-allocated (diverted away from timing), each clock is unable to maintain its current subjective time in WM, and the response is delayed [16], [17], [27] in proportion to the perceived salience of the distracter relative to the times signal [34], [35]. Indeed, the effect of a gap is affected by the contrast in intensity between the gap and the timed signal [27], [34], [39] and by the perceptual acuity of the subjects [27]. Importantly, the predictions of this resource re-allocation model are not restricted to retention-intervals (i.e., gaps), but extend to distracters presented during the continuous presentation of a timed signal. Previous data were quantitatively accounted for by assuming that during a gap and/or distracter the accumulated time decays at a rate that varies with the relative salience of the distracter to the timed signal [25], [26], [35]. Here we found that the comparison between the gap and the (multiple) timed signal(s) extends to the temporal domain. Simulations indicate that both the differential effect of the gap on multiple durations, and the gradual hierarchical reset due to increasing gap duration can be quantitatively accounted for assuming that the three clocks have separate resources re-allocated independently during a gap at a rate proportional to the ratio *g*/*T_k_* between the duration of the gap and each criterion duration (Fig. 2A:D-Model).

### Implications for Neurobiological Models of Interval Timing

Our finding that timing involves multiple independent clocks has direct biological support from studies examining activation firing in the striatum (STR) and prefrontal cortex (PFC) in timing tasks [3]. When rats are probabilistically rewarded at two durations, distinct subsets of STR neurons are activated for the two temporal criteria [40]. Similarly, in the present task the three criteria may have been encoded by independent STR neural populations, differentially affected by the presentation of the gap. Importantly, STR neurons can code multiple durations only if PFC is intact. Lesions of the agranular frontal cortex (AgFC) or the nucleus basalis magnocellularis [8] impair rats' ability to time two stimuli simultaneously, but not the ability to time each stimulus sequentially [41]. Moreover, AgFC is implicated in divided attention to multiple timed stimuli [42], and in the dynamic allocation of attention in time [43]. These results are compatible with our finding that gaps inserted into the signal differentially affected the timing of the multiple temporal criteria by the means of re-allocation of attentional or memory resources.

The above information is embedded in a neurobiological model of interval timing – the Striatal Beat Frequency (SBF) model – which describes interval timing as an emergent activity in the cortico-striatal circuits based on the coincidental activation of medium spiny neurons in STR by cortical neural oscillators [44], [45]. Experience-dependent changes in cortico-striatal transmission are assumed to make STR neurons more likely to detect the pattern of activation of cortical oscillators at the time of reward [46]. In the SBF model, the activity of STR neurons increases before the expected time of reward, and peaks at the criterion interval, a result that parallels ensemble recordings of STR neurons in time reproduction procedures [40], and behavior in the present study (Fig. 1A). Moreover, the SBF model demonstrates the scalar property [44], i.e., the widths of the response functions increase with the criterion duration, a result evident in our experiment (Fig. 1A). In the framework of the SBF model, the current study suggests that the different STR neural populations, which independently code for multiple durations, are driven by separate pools of cortical oscillators which can be differentially stopped and/or reset.

### Conclusions

We found that events are judged relative to the temporal contexts in which they occur. Multiple temporal contexts are coded simultaneously by multiple internal clocks that can be independently ran, stopped, or reset by the insertion of a gap into the signal. Quantitative modeling suggests that the multiple clocks have separate attentional or working memory resources, which are re-allocated independently during a gap at a rate proportional to the relative duration of the gap to the multiple criterion durations. These findings have direct support from studies of neural firing during timing studies showing that timed criteria are encoded by separate neural populations, possibly activated by independent populations of cortical oscillators, which can be differentially stopped/reset based on relative activity in STR and PFC. These data extend the applicability of a relative theory of temporal categorization involving space and time [22], [47] to multiple contexts. This perspective suggests that our brain is continuously timing multiple events within different temporal contexts, and represents the durations of these events not in an absolute manner, but relative to their contexts [48].

## Materials and Methods

### Subjects

Ten male Sprague-Dawley rats (Charles-River Labs, Raleigh, NC) approximately 4 months of age at the beginning of the experiment were used as subjects. Rats were given continuous access to water and maintained at 85% free-feeding weight by a daily ration of rat chow (Rodent Diet 5001, PMI Nutrition International, Inc., Brentwood, MO).

### Apparatus

The apparatus consisted of 10 standard lever boxes designed for rats (MED Associates, Inc., St. Albans, VT) housed in sound attenuating cubicles. The lever boxes were equipped with three response levers (two retractable and one fixed) situated on the front wall. According to the schedule, 45-mg precision food pellets (Bioserve, Frenchtown, NJ) were delivered in a food cup situated on the front wall, 1 cm above the grid floor, under the center lever, by the pellet dispenser. The to-be-timed stimulus was a 28-V 100-mA house light mounted at the center-top of the front wall. A 66-dB background sound produced by a ventilation fan was present throughout the session.

### Procedure

Rats learned to time multiple target durations using a version of the tri-peak procedure [11], [12], [18]. Briefly, rats were trained with three types of fixed-interval (FI) trials, interspersed randomly. Trials began with the illumination of the house light. The first response after the appropriate duration (10, 30, or 90s) was reinforced with a 45 mg food pellet and the house light was turned off for a random ITI (60±30s). Following FI training, non-reinforced probe trials, which lasted 3 times the 90-s criterion time plus a random 0–20% (270–330s), were added to the sessions. During testing, four non-reinforced gap trials were added to the trial types randomly selected for. Gap trials lasted for the same length as probe trials, except that gaps of different durations (1, 3, 10, or 30 s) were inserted into the timed signal at a constant location (15s from trial onset).

### Response Rate Analysis

The time of each lever press was recorded in 1-s bins. Response functions were separately fitted by linear functions and by Gaussian + ramp functions using SigmaPlot (SPSS, Chicago, IL). The mean of the fitted function was used as a measure of the peak time [39]. The ratio of the r^2^ values from the linear and Gaussian + ramp function was used as a measure of the temporal control exhibited by each rat. If this ratio was greater than 0.8, the rat's data for that criterion duration was not used because they do not provide useful information regarding the rat's temporal perception. Less than 2% of the data were excluded for this reason.

### Individual-Trials Analysis

The *s_1_*, *s_2_*, *d*, and *m* parameters for each trial were estimated for each criterion as described by Church et al [19], and correlations among these parameters were computed for each target duration. Responses on the short, medium, and long levers from and the 90-s probe trials (in which the rat responded on all three levers without reinforcement) were fit separately. For within-duration correlations, only trials with *s_1_* falling prior to the obtained target duration, and *s_2_* falling after the target duration were used, in order to avoid forcing positive correlations [19]. For similar reasons, for between-duration correlations only trials in which the *s_2_* for one target duration did not overlap with the *s_1_* of the next target duration were used. Fewer than 10 percent of the trials were excluded on this basis.

### Quantitative Modeling

Quantitative modeling was conducted assuming three independent “pacemaker-switch-accumulator” clocks of criteria *T_k_, k = 1, 2, 3,* which provide three independent representations of the subjective time. Clocks were assumed to have a common pacemaker but independent switches and accumulators. Subjective time was assumed to accumulate independently in the three accumulators before and after the gap at a fixed unitary rate for all clocks, but decay exponentially during the gap at different (independent) rates, *salience* /*T_k,_* where *salience* is the relative salience of the gap to the timed signal (considered constant in the present simulations). Parameters used in all simulations: *pacemaker rate*  = 1, *salience*  = 4, *T_1_ = 10*,* T_2_ = 30*, *T_3_ = 90*.
